# Tuning Crystal Growth of Colloidal Cs_3_Bi_2_I_9_ Perovskite‐Like Nanocrystals via a Solvent‐Assisted Reprecipitation Approach

**DOI:** 10.1002/cssc.202501957

**Published:** 2025-11-06

**Authors:** Valentina Bellotti, Francesca Pallini, Sara Mattiello, Charl Xavier Bezuidenhou, Clara Saetta, Giovanni Di Liberto, Vanira Trifiletti, Abdolhamid Khodadadi, Gloria Zanotti, Roberto Flammini, Giorgio Contini, Luca Beverina

**Affiliations:** ^1^ Department of Material Science Università degli Studi di Milano‐Bicocca Via Roberto Cozzi 55 20126 Milano Italy; ^2^ Istituto di Struttura della Materia CNR (ISM‐CNR) Via del Fosso del Cavaliere 00133 Roma Italy; ^3^ Istituto di Struttura della Materia CNR (ISM‐CNR) Strada provinciale 35d/9 00010 Montelibretti Italy

**Keywords:** colloidal synthesis, electrostatic stabilization, lead‐free perovskite, solvent engineering

## Abstract

Lead‐free bismuth‐based perovskite‐like materials have emerged as promising alternatives to lead halide perovskites due to their enhanced stability and lower toxicity. Colloidal approaches for the synthesis of such materials offer the advantage of morphology engineering and precise control over size and shape. However, conventional approaches require long‐chain organic ligands, which hinder charge transport and limit their applicability in optoelectronic devices. Herein, a ligand‐free Solvent‐Assisted Reprecipitation (SARP) approach is introduced, exploiting solvent‐mediated synthesis to control the nucleation and growth of Cs_3_Bi_2_I_9_ nanocrystals. The use of solvent descriptors, such as Kamlet–Taft parameters, enables preliminary screening of suitable solvents, suggesting five key candidates, including the green solvents triethyl phosphate and Cyrene, best suited to control crystal size, aspect ratio, and morphology. Electrostatic stabilization, induced by the solvent, promoted the formation of small Bi^0^ islands at the surface, which is key to ensuring colloidal stability and processability. Computational and experimental analyses agree on highlighting the role of solvents in controlling nucleation and growth of the nanocrystals, paving the way for the rational design of ligand‐free nanomaterials for a platform of different applications.

## Graphical abstract

A ligand‐free, solvent‐assisted route enables precise control of Cs_3_Bi_2_I_9_ nanocrystals via solvent‐mediated crystal nucleation. Guided by Kamlet–Taft descriptors, green candidates such as triethyl phosphate and Cyrene, along with reference solvents dimethylformamide and dimethyl sulfoxide, to tune size, shape, and colloidal stability for scalable lead‐free materials.

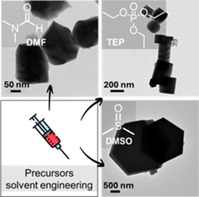

## 1 Introduction

Main text paragraph. Lead halide perovskite nanocrystals (LHP‐NCs) have gained immense attention in recent years due to their exceptional optoelectronic properties, making them very appealing in a wide variety of technologies such as luminescent solar concentrators,^[^ ^1^ ^]^ solar cells,^[^ ^2^, ^3^ ^]^ light‐emitting diodes,^[^ ^4^, ^5^ ^]^ and scintillator detectors.^[^ ^6^, ^7^, ^8^ ^]^ Despite their performances, a poor long‐term stability^[^ ^9^ ^]^ and environmental concerns associated with lead have driven the scientific community to study alternatives,^[^ ^10^, ^11^ ^]^ aiming at improving stability and safety without eroding efficiency. Among various alternatives, lead‐free perovskite‐like structures have been synthesized using Sn, Sb, Bi, Ge, and others.^[^ ^12^, ^13^, ^14^ ^]^ Even though Sn and Ge belong to the same group as Pb, their high oxygen sensitivity may lead to instability and alteration of their properties through metal ion oxidation. In addition, their toxicity remains relevant to human health. For these reasons, the more environmentally stable and less toxic VA group metals (Bi and Sb) have been considered as lead replacement and tested for photovoltaics, X‐ray detection, and thermoelectric applications.^[^ ^15^, ^16^, ^17^ ^]^

Similar to APbX_3_ LHP‐NCs, lead‐free alternatives have been synthesized using both hot‐injection (HI)^[^ ^18^, ^19^, ^20^ ^]^ and ligand‐assisted reprecipitation (LARP) methods.^[^ ^21^, ^22^ ^]^ As reported, the HI method presents limitations in terms of scalability and absolute yield, which have been partially addressed by the LARP technique.^[^ ^23^, ^24^ ^]^ However, both colloidal approaches rely on crystal stabilization mediated by ligands featuring long alkyl chains, such as oleic acid and oleyl amine, anchored on the newly formed perovskite surface. While these ligands play a crucial role in stabilizing the nanocrystals, they can be highly detrimental for applications requiring efficient charge transport, as they introduce insulating barriers, hinder carrier mobility, and promote the formation of trap states, ultimately limiting performance.^[^ ^25^, ^26^, ^27^ ^]^ For this reason, the development of ligand‐free colloidal strategies is appealing, albeit still challenging. Among various parameters influencing perovskite synthesis, solvent selection plays a crucial role in determining crystallinity and morphology by tuning crystal growth kinetics and nucleation. Recently, Abate et al.^[^ ^28^ ^]^ systematically investigated the role of solvents in the synthesis of formamidinium tin iodide (FASnI_3_) perovskites, demonstrating how the choice of the solvent can prevent precursor oxidation, leading to improved solar cell performance. Similarly, extensive research has been carried out on solvent effects in lead‐based perovskites.^[^ ^29^, ^30^, ^31^ ^]^ However, no such systematic studies have been reported so far for bismuth‐based perovskites.

**Scheme 1:**
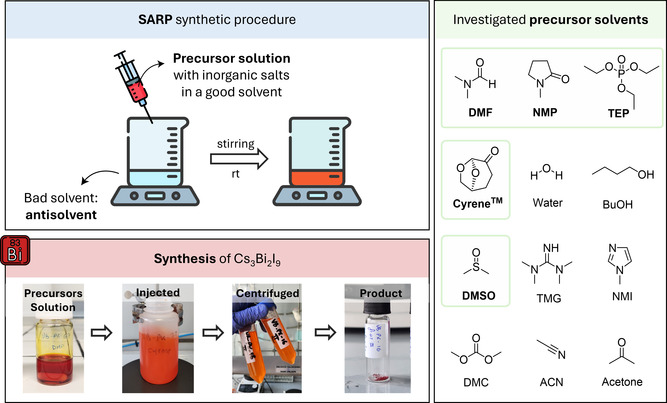
Schematic representation of SARP synthesis at mild ambient conditions using different precursor solvents. Precursor solutions are all injected into toluene, centrifuged, and the recovered powder is dried. In the first panel, “good solvents” refers to solvents able to solubilize and stabilize the Cs and Bi precursors (precursor solvents) whereas “bad solvents” refers to solvents able to induce the crystal growth and precipitation (antisolvent). Solvents leading to the formation of the target material are highlighted with green squares (dimethyl sulfoxide (DMSO), N,N‐dimethyl formamide (DMF), Triethyl phosphate (TEP), Cyrene, and N‐Methyl‐2‐pyrrolidone (NMP)).

Here, we describe a solvent‐assisted reprecipitation (SARP) method (**Scheme** **1**) for a colloidal synthesis of Cs_3_Bi_2_I_9_ (CBI) nanocrystals. The choice of solvent is critical to control size and morphology and is guided by two key factors: 1) classification based on Kamlet–Taft (KT) solubility parameters^[^ ^32^ ^]^ and 2) the redox behavior of the solvent or its byproducts. Based on the KT parameters, five promising candidates, including green solvents, were identified. The chemical properties of the solvents help explain trends in crystal size and shape. Computational and experimental analyses were used to explore how the solvent‐specific relative stabilization of precursors, small clusters, and crystals of the target phase impacts dimensions and superficial charge, leading to electrostatic stabilization and colloidal stability. Our findings help shed light on the properties of bismuth‐based perovskites, opening the way to the development of sustainable and efficient lead‐free next‐generation materials for a wide variety of applications.

## 2 Results and Discussion

### 2.1 Precursors Stabilization: KT Solubility Parameters

Solubility parameters are valuable tools for quantitatively classifying and predicting the relative solvency behavior of solvents. In fact, the ability of a solvent to solubilize the inorganic precursors CsI and BiI_3_ represents a crucial initial step for the synthesis of ligand‐free CBI. Rather than relying on the more common Hildebrand and Hansen approaches, we selected the KT parameters, due to the additional advantage of accounting for the solvent polarizability (π*), a key factor when working with inorganic salts as precursors (additional information in section S1, Supporting Information).

**Figure 1:**
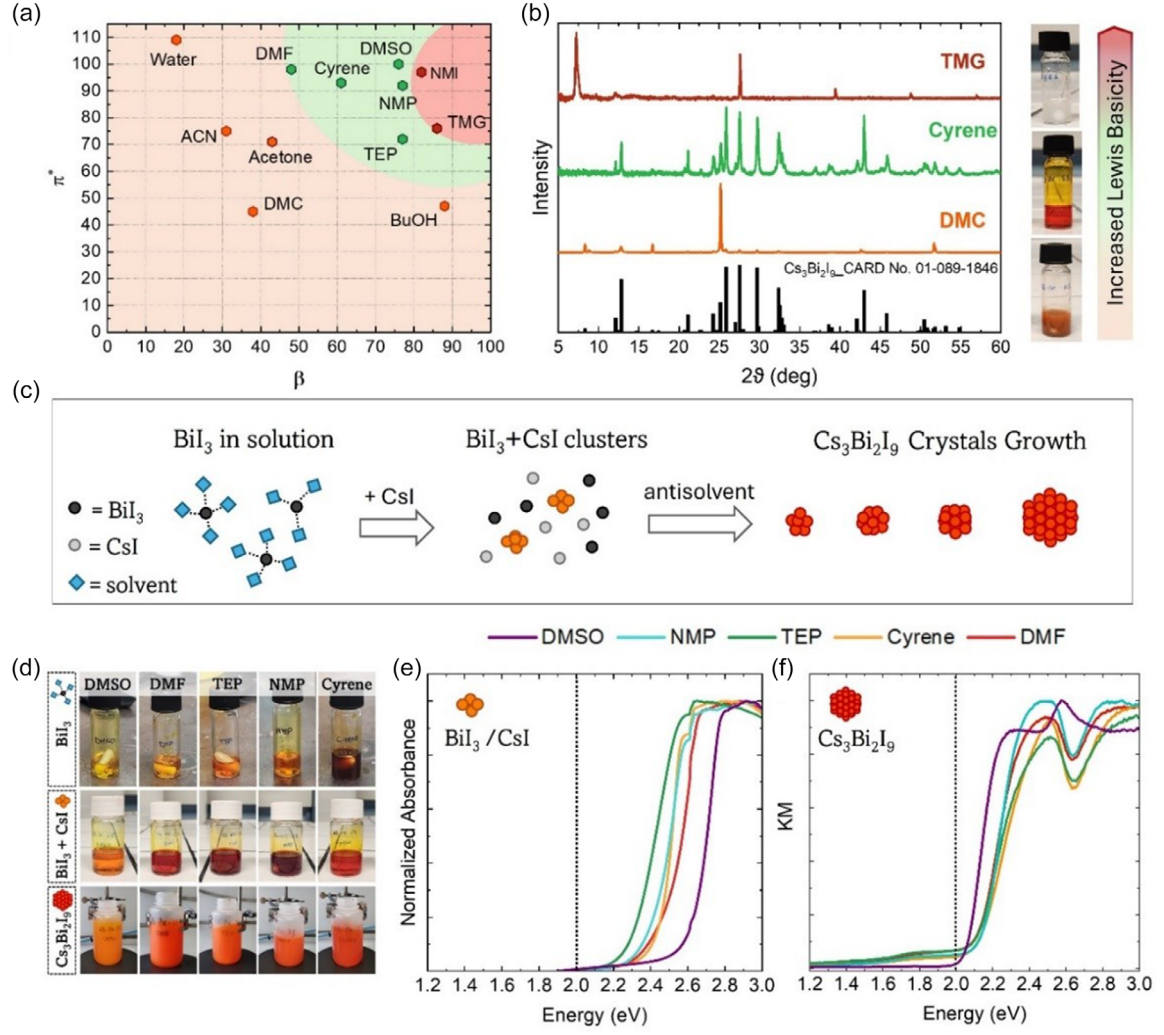
a) KT plot representing the solvent's polarizability (π*) and hydrogen bond acceptor ability (*β*). The colored regions were added as a guide for the eyes. The red region includes solvents that do not produce Cs_3_Bi_2_I_9_ crystals, whereas for solvents inside the orange region, CBI forms in an uncontrolled way, due to the inability of the solvents to dissolve the precursors. The green region includes the solvents that solubilize the precursors and allow CBI formation upon injection in an antisolvent. b) XRDs of samples produced by reprecipitation from a solvent for each of the three colored regions (green, orange, and red). The tetramethylguanidine (TMG) sample depicted by the red line shows only peaks related to CsI, whereas dimethyl carbonate (DMC), represented by the orange line, shows peaks attributed to both CBI and CsI phases. c) schematic illustration and d) pictures of the SARP synthesis steps, starting from BiI_3_ solubilization, going to CsI addition and cluster formation, and finishing in the crystal growth after injection in the antisolvent. Absorption spectra of e) BiI_3_/CsI clusters in DMF, DMSO, TEP, NMP, and Cyrene, and f) of CBI powder measured with an integrating sphere and obtained from the same five solvents. The signal saturation in (e) is due to the necessity of measuring the cluster solution without altering the precursor concentration, due to thermodynamic equilibrium issues explained in Figure S2, Supporting Information.

As such, we selected various solvents to probe the KT space (all parameters are reported in Table S1, Supporting Information). By considering π* and the hydrogen bond accepting capability (*β*), a clear pattern emerges, as highlighted in **Figure** **1**a. Perovskite formation is not favored for both very large and small values of *β*. More precisely, high Lewis basicity (*β *> 80) implies a strong interaction of the solvent with the precursors, resulting in no perovskite formation as shown by the X‐ray difraction (XRD) pattern (Figure 1b, **red line**). On the other hand, when the *β* value is low (*β* < 45), the solvent does not allow efficient solubilization of CsI and BiI_3_, leading to partial conversion in the target phase on the surface of the suspended precursor particles even without injection in the antisolvent. This effect is observed in the XRD (Figure 1b **, orange line**), where both peaks of CBI and CsI are present.

A similar pattern is observed when the solvent polarizability (π* parameter) decreases, so that only solvents having intermediate values of both parameters (70 < π* < 100 and 45 < *β* < 80) can efficiently dissolve both CsI and BiI_3_ precursors, enabling perovskite formation upon injection into an antisolvent. The five solvents considered in this study are contained in the green area in Figure 1a and are represented in Scheme 1. These include the popular dimethyl sulfoxide (DMSO), N,N‐dimethyl formamide (DMF), and N‐Methyl‐2‐pyrrolidone (NMP), as well as the greener alternatives triethyl phosphate (TEP), and Cyrene. Remarkably, KT parameters help classify and select suitable solvents for the formulation of inks of the nanocrystals obtained with our SARP protocol. (Figure S1, Supporting Information).

Similar to lead halide perovskite, we hypothesize that Cs_3_Bi_2_I_9_ crystal formation occurs by the preliminary formation of BiI_3_/CsI clusters, which can be stabilized differently depending on the solvent (Figure 1c). In fact, the addition of CsI into the BiI_3_ solution causes a color change from colorless to orange/red (Figure 1d). The formation of BiI_3_/CsI clusters relies on an equilibrium process that strongly depends on the concentrations of the two species, as shown in the Supporting Information (Figure S2, Supporting Information). The shape and maximum wavelength of the UV–vis absorption spectrum of clusters obtained in DMSO (Figure 1e) are the closest to those of pure BiI_3_ (Figure S2a, Supporting Information), whereas in the other solvents, the absorption maximum gradually shifts to resemble that of CBI after injection into toluene (Figure 1f). It is already known that DMSO shows strong coordinating ability with respect to BiI_3_, enabling the formation of a stable BiI_3_(DMSO)_2_ complex.^[^ ^33^ ^]^ This strong interaction could be the reason why crystal nucleation in DMSO is less efficient, and the absorption of the precursors is more similar to that of pure BiI_3_. On the other hand, the cluster formation and crystal nucleation in the other precursor's solvent is more efficient, and the absorption properties of BiI_3_/CsI clusters resemble those of CBI crystals. The formation of a larger number of seeds results in smaller crystals with increased surface area. This trend is confirmed by the morphological characterization in the following sections.

### 2.2 Solvents Reactivity: Redox Properties

**Figure 2:**
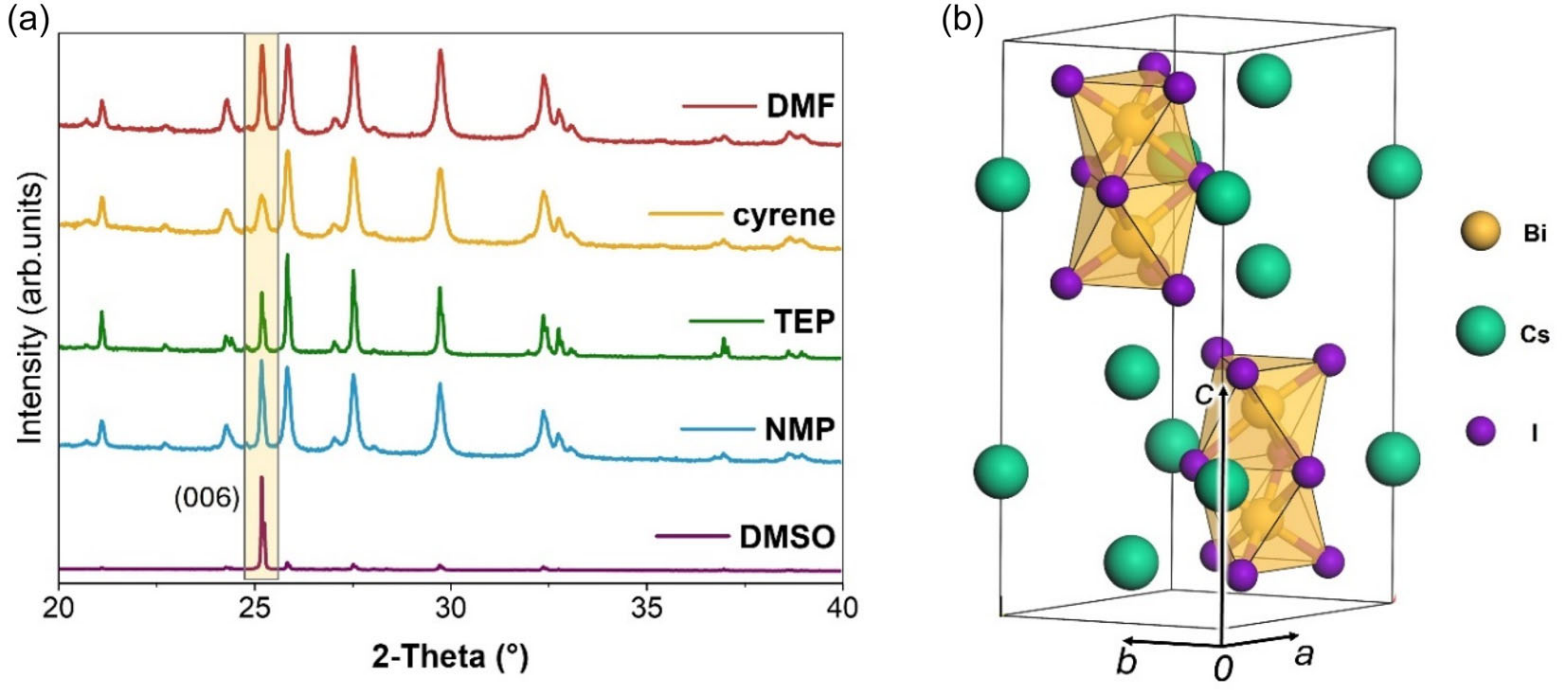
a) XRD pattern of the five samples with normalized intensity. The yellow rectangle highlights the 006 crystal plane whose relative intensity varies as a function of the sample (*λ *= 1.54449 Å). b) Perspective view of the crystal structure of Cs_3_Bi_2_I_9_.

**Figure 3:**
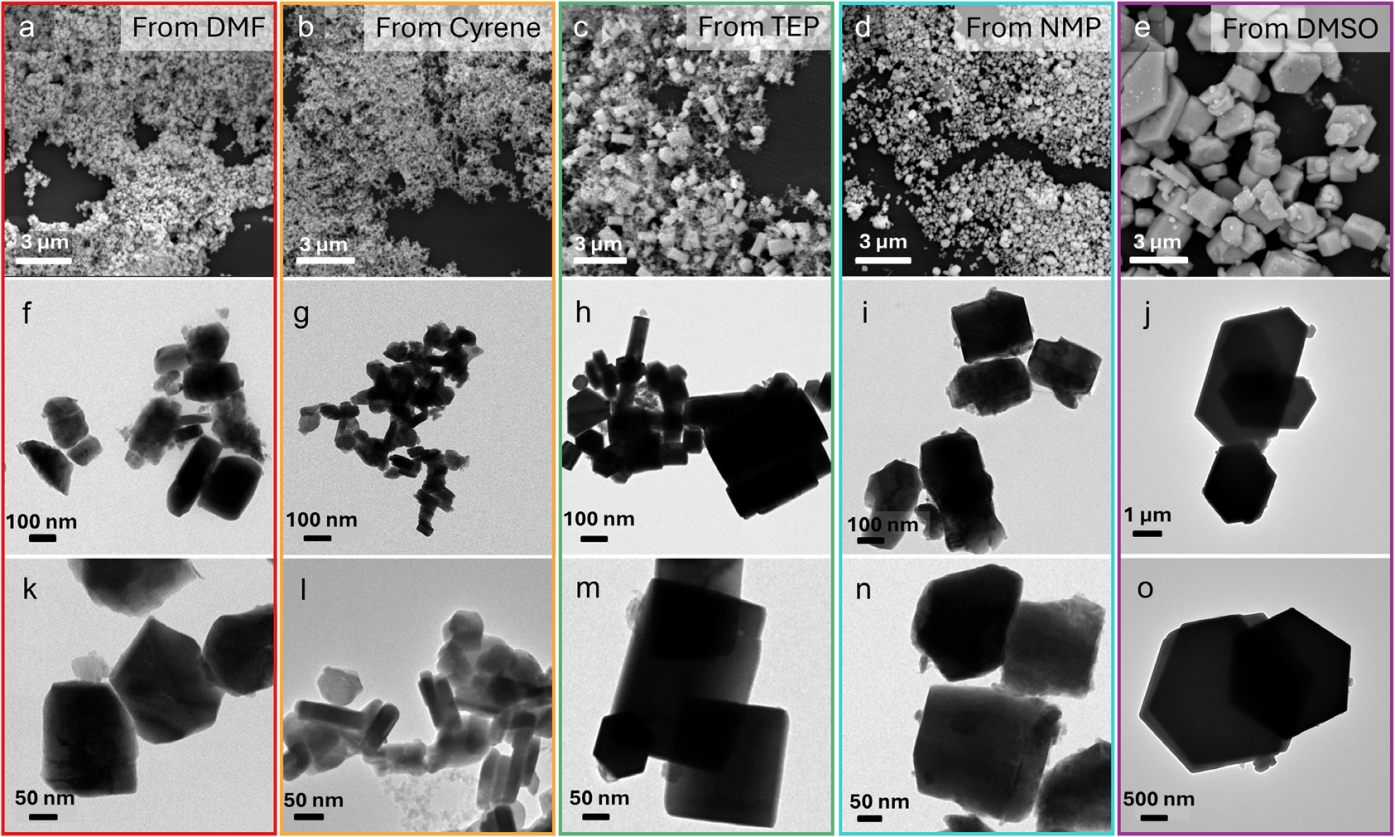
On the first line, SEM images of samples derived from a) DMF, b) Cyrene, c) TEP, d) NMP, and e) DMSO. On second‐ and third‐lines TEM images respectively of: f,k) DMF, g,l) Cyrene, h,m) TEP, i,n) NMP, j,o) DMSO.

The KT parameters help select solvents that are likely to dissolve the precursors. However, this factor alone is not sufficient to predict the features of the obtained nanocrystals. Although all five samples exhibit electron energy gaps (*E*_g_ ≈ 2.1 eV), thermal stability (Figure S3, Supporting Information), and UV–vis absorbance features that are consistent with the literature, they differ in the preferential crystal orientations (**Figure** **2**), as well as size and morphology (**Figure** **3**).

Notably, using DMSO results in micrometer‐sized hexagonal crystals that closely resemble CBI single crystals.^[^ ^34^ ^]^ On the other hand, DMF, Cyrene, and NMP yield crystals smaller than 130 nm, while larger objects are formed with TEP and DMSO (see dimensional analysis, Figure S4, Supporting Information). Interestingly, not only the size of the crystals, but also their morphology differs as a function of the precursor solvent. The energy dispersive X‐ray analysis (Figure S5, Supporting Information) mapping of Cs, Bi, and I in the samples shows homogeneous composition.

The different solvents impact on the XRD patterns (Figure 2a). In particular, CBI synthetized from DMSO, TEP, and NMP shows sharper signals, with peaks splitting in the case of TEP and DMSO. Both features hint at high crystallinity and large crystallite dimensions. On the other hand, samples obtained using DMF and Cyrene show the broad reflections typical of nanocrystals. The average grain size of the crystals calculated by Rietveld refinement plot follows the same trend observed by Transmission electron miscroscopy (TEM) analysis, with an average size for Cyrene and DMF of 49 and 71 nm, and 130 nm for NMP (Table S2, Supporting Information). Furthermore, the preferred orientation observed for CBI grown from DMSO was quantified by Rietveld refinement using the March–Dollase approach (Figure S6, Supporting Information).^[^ ^35^ ^]^ The March parameter was refined for the (0 0 6) reflection (*r *= 0.435(4)), which yielded a percentage degree of preferred orientation (*η*) of 60%.

The formation of nano‐ rather than microcrystals, without ligand‐enforced surface stabilization, is nontrivial. According to the general principles of colloidal stabilization, electrostatic repulsion is the most likely explanation. We speculated that solvent‐induced surface modification could be responsible for the presence of net charges on the surface, preventing coalescence and limiting the growth. It is well known that Bi(III) compounds can be reduced to Bi^0^ (metallic bismuth) in the presence of suitable reducing agents such as NaBH_4_,^[^ ^36^ ^]^ hydrazines,^[^ ^37^ ^]^ and even alcohols under transfer hydrogenation conditions.^[^ ^38^ ^]^ The formation of metallic Bi clusters on the perovskite surface would lead to bismuth cation vacancies and thus to overall negative charging of the surface. The ligand‐free CBI featuring nanoscale crystals (below 130 nm) showed good colloidal stability over days, comparable with ligand‐stabilized CBI synthetized via LARP (Figure S7, Supporting Information).

Experimental techniques, such as X‐ray photoelectron spectroscopy (XPS) and NMR, as well as density functional theory (DFT) simulations, fully support our interpretation.

**Figure 4:**
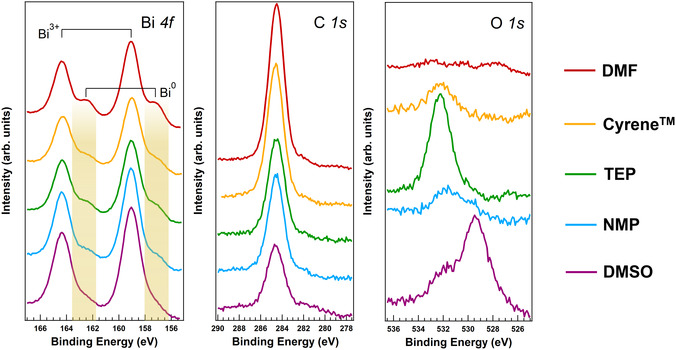
Core level XPS spectra of Bi 4f, C 1s, and O 1s regions for Cs_3_Bi_2_I_9_ processed from different solvents, including DMF, Cyrene, TEP, NMP, and DMSO. Peaks associated with Bi^3+^ and Bi^0^ are highlighted with a yellow rectangle.

**Figure** **4** presents the core‐level spectra of Bi 4f, C 1s, and O 1s obtained from CBI crystals synthesized in various solvents (refer to Section S3 of the Electronic Supporting Information for the survey scan and peak deconvolution details). The spectra exhibit peaks at binding energies (BE) of 159.0 eV and 164.3 eV, corresponding to the Bi 4f_7/__2_ and Bi 4f_5/__2_ levels, respectively, and are characteristic of Bi^3^^+^ species. In addition to these main peaks, shoulders at 157.2 eV and 162.5 eV—indicative of metallic bismuth (Bi^0^)—are observed, with their relative intensities varying depending on the solvent used.

As shown in Table S3, Supporting Information, the Bi^0^/Bi^3^^+^ intensity ratio follows the order DMF > Cyrene ≈ TEP > NMP ≈ DMSO, reflecting the reducing potential of each solvent. While DMSO is not typically reported as a reducing agent in the literature, it is frequently involved in oxidative transformations, such as the Swern oxidation of secondary alcohols to ketones.^[^ ^39^ ^]^ The growth of CBI crystals in DMSO yields large crystals with minimal Bi^0^ contributions in the XPS spectra. Conversely, DMF is known to decompose readily into carbon oxides (CO*_X_*) and dimethylamine (DMA),^[^ ^40^ ^]^ the latter being a well‐established reducing agent used in the synthesis of metal nanoparticles.^[^ ^41^ ^]^ NMR analysis (Figure S11, Supporting Information) confirmed the presence of appreciable amounts of DMA in the DMF batch employed. Consistent with this observation, DMF led to the formation of CBI nanocrystals exhibiting the most intense Bi^0^ signal in the XPS analysis.

As for the other solvents we used, cyclic acetals are known to be reducing solvents, and Cyrene in particular has already been used to produce Bi^0^ nanoparticles as reported by Hernández–Pagán et al.^[^ ^42^ ^]^ Finally, TEP can be hydrolyzed in phosphoric acid and ethanol^[^ ^43^ ^]^ (as shown by ^1^H NMR in Figure S12, Supporting Information), activating transfer hydrogenation pathways.

The XPS analysis of O 1s and C 1s levels shown in Figure 4 gives additional insight into the mechanism. The presence of carbon peaks is usually negligible in intensity for Cs_3_Bi_2_I_9_ perovskites prepared via spin coating methods. However, in all five samples, carbon has been detected with intensity decreasing from DMF to DMSO, in pairs with the changes in morphology we already discussed (Figure S6, Supporting Information). Interestingly, when it comes to oxygen, the peak intensity of the DMF sample is much lower than expected based on the C 1s trend. This result validates the hypothesis of the amide decomposition into amine, leading to growth under reducing conditions. The deconvolution of the O 1s core‐level spectra shows two main peaks at BE = 532.2 eV and BE = 529.7 eV (Figure S10, Supporting Information). The peak at the higher binding energy is linked to oxygen vacancies^[^ ^44^ ^]^ and hydroxyl groups, including bridging hydroxyls.^[^ ^45^ ^]^ The lower binding energy component can be assigned to metal–oxygen interactions, specifically O^2−^ in bismuth oxide (Bi—O bonds).^[^ ^46^, ^47^, ^48^ ^]^ These results indicate a combination of intrinsic lattice oxygen and surface‐adsorbed species, with variations in peak intensities reflecting differences in oxygen defect concentrations.

**Figure 5:**
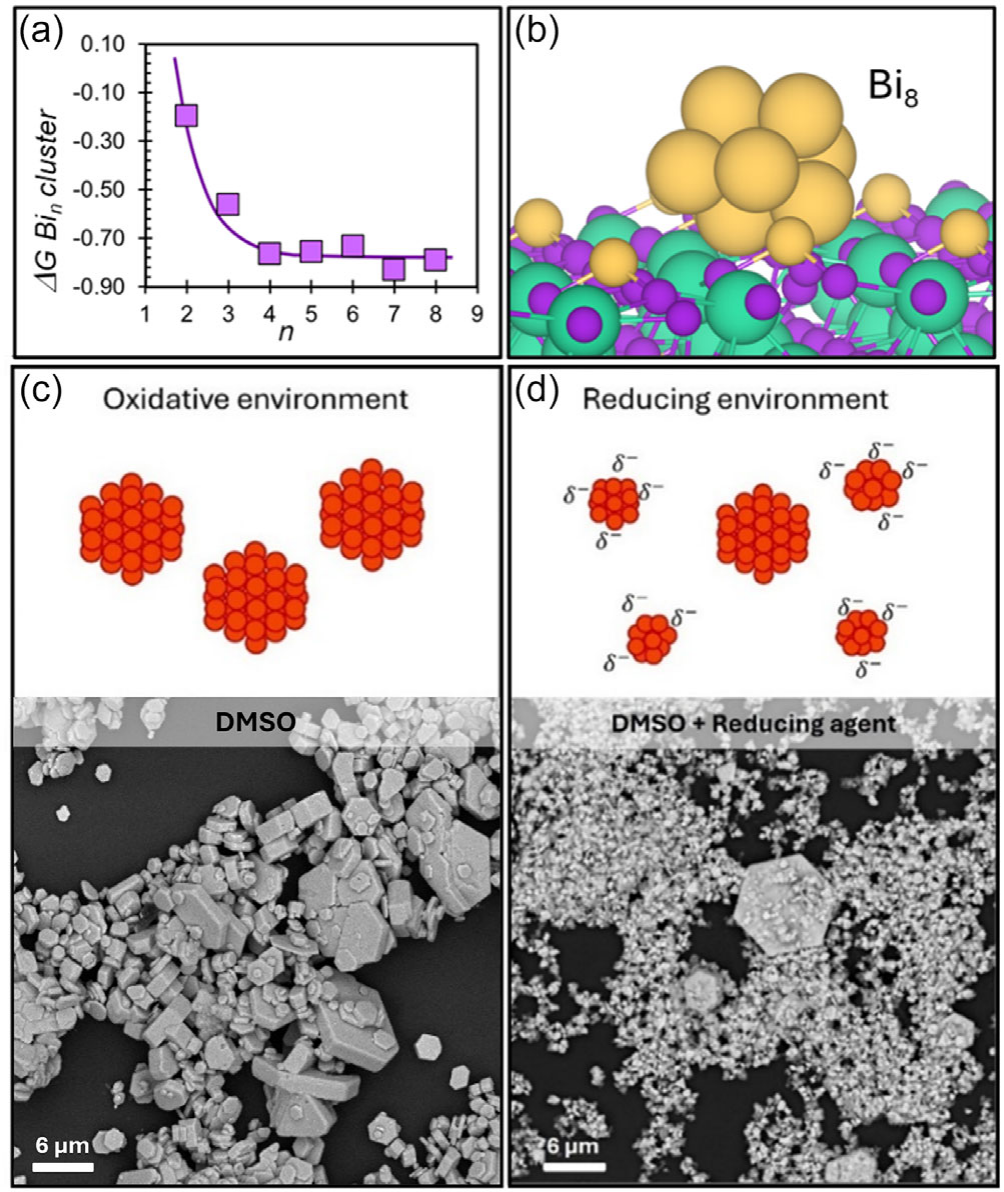
a) Reaction free energies (in eV) for the Bi_n_ cluster formation normalized by the number of atoms; b) Bi_8_ clusters adsorbed on the surface of Cs_3_Bi_2_I_9_ (Cs: green; Bi: yellow; I: purple). On the top schematic representation and on the bottom SEM images of nanocrystals obtained in, c) oxidative environment in DMSO, and d) reducing environment in DMSO with the addition of phenyl hydrazine.

The process leading to the formation of metallic Bi aggregates on the CBI crystal surfaces was further investigated through DFT simulations. First, we performed a systematic assessment of the stability of different terminations of the low‐index (001) surface, where the atoms cut by the reticular plane are Bi, I, and CsI_3_ units. Results show that Bi‐ and I‐ terminated have comparable surface energies, 0.48 Jm^−2^ and 0.47 Jm^−2^, respectively (Figure S13 and Table S5, Supporting Information). These models were used for further investigations, as detailed below. As a case study, we focused on nanocrystals formed in DMF to understand the key interactions at the material surface and the associated redox pathways. As previously mentioned, we hypothesized that the presence of DMA, resulting from DMF decomposition, generates a reducing environment capable of generating Bi^0^ islands at the surface. Our calculations neglect implicit solvation, as the purpose here is to understand the nature of the interaction between the material structure and DMA. Also, we considered just a single molecule as the explicit simulation of a solvation environment would result in an unaffordable computational cost. Despite the inherent approximations of the model, the general trend should be consistent. Our computational model predicts that the interaction between DMA and surface atoms of CBI crystals is energetically favorable (Figure S14a,b, Supporting Information). In particular, DMA shows strong binding affinity toward Bi species, with an adsorption free energy of −4.27 eV, whereas adsorption onto Cs exposed sites is less favorable, with an adsorption free energy of −2.78 eV. DMA coordinates the metal center with the lone pair localized on the nitrogen. The reduction of Bi^3+^ to Bi^0^ upon coordination is accompanied by the oxidation of DMA to Me_2_NO. **Figure** **5**a shows the reaction Gibbs energies for the formation of the Bi_n_ cluster as a function of the number of constituent atoms, normalized per Bi atom. For clusters consisting of at least 4 atoms, the energy gain converges to a nearly constant value, supporting the spontaneous growth of metallic Bi clusters as observed experimentally. The Gibbs free energy for the formation of a Bi_4_ cluster and oxidized DMA is –0.16 eV, suggesting that solvent–surface interactions can drive Bi aggregation. Similarly, the formation of Bi_8_ (Figure 5b) is associated with the oxidation of two DMA molecules and a Gibbs free energy of –0.53 eV. It is noteworthy that the trends predicted in vacuum align with the experimental observations. While solvent effects may shift absolute free‐energy values, they are not expected to invert the overall stability trend of cluster formation. In short, the process of formation of Bi^0^ clusters at the surface in the presence of DMA is spontaneous and results in a net negative residual charge, which is responsible for the colloidal stabilization we observe.

To further support our interpretation, we repeated the synthesis of the CBI crystals in DMSO, but also included phenyl hydrazine as a reducing agent. As shown in Figure 5d, we obtained a result that is fully consistent with what we previously observed working with DMF.

In short, our method provides a tool to control the morphology and colloidal stability of ligand‐free Bismuth‐based perovskite materials, solely based on the informed selection of the precursor's solvent. Rather than comparing with other ligand‐free methods developed for the synthesis of nanocrystals with specific characteristics, we offer a general tool to map the whole potential space of different sizes and morphologies. Moreover, the SARP technique is easily scalable, as confirmed by targeting 1 g of CBI material (see Section S6, Supporting Information). The use of a turbo‐emulsifier allowed for the fast homogenization of 1 L nanocrystal dispersion, further confirming the robustness of SARP methodology.

## 3 Conclusion

We reported a novel ligand‐free synthetic approach for the preparation of Cs_3_Bi_2_I_9_ perovskites via SARP, employing solvent engineering to finely control crystal growth under mild conditions. A systematic solvent classification based on KT parameters facilitated the initial screening of five effective solvents, spanning conventional to environmentally benign options, capable of dissolving the precursors efficiently and enabling tunable modulation of crystal size and morphology. Very few attention has been given to the use of green solvents for Bi‐based perovskite‐like materials; for this reason, this could be a starting point for the studies of other green alternatives in this field.

Our findings highlight that, beyond solvation ability, the redox characteristics of the solvents critically influence the formation of Bi^0^ species, which in turn govern the final nanocrystal size and surface properties. The resulting surface charge imbalance induces electrostatic stabilization, allowing the formation of stable nanocrystals in the absence of insulating organic ligands, thus addressing a key limitation of traditional colloidal syntheses. A comprehensive combination of experimental techniques (XPS, SEM/TEM, XRD) and DFT simulations substantiates the occurrence of solvent‐induced Bi^3^^+^ reduction and its impact on nucleation and crystal growth dynamics. This study not only advances the fundamental understanding of solvent effects in bismuth‐based perovskite systems but also establishes a sustainable route toward ligand‐free nanomaterials, giving precise guidelines in the solvent selection, according to the desired crystal size and morphology for the chosen application.

## Supporting Information

The authors have cited additional references within the Supporting Information.^[^ ^8^, ^24^, ^32^, ^35^, ^49^, ^50^, ^51^, ^52^, ^53^, ^54^, ^55^, ^56^, ^57^, ^58^, ^59^, ^60^, ^61^, ^62^, ^63^, ^64^, ^65^, ^66^, ^67^, ^68^, ^69^ ^]^

## Conflict of Interest

The authors declare no conflict of interest.

## Accession Codes

CCDC 2494290‐2494294 contain the supplementary crystallographic data for this paper.

## Supporting information

Supplementary Material

## Data Availability

The data that support the findings of this study are available in the supplementary material of this article.
