# Supplementary material for: Tuning Crystal Growth of Colloidal Cs3Bi2I9 Perovskite‐Like Nanocrystals via a Solvent‐Assisted Reprecipitation Approach
Source: ChemSusChem. 2025 Nov 6;18(24):e202501957. doi: 10.1002/cssc.202501957 (PMC12703447; doi:10.1002/cssc.202501957)
Supplement: Supplementary file 1 — Supplementary Material [file CSSC-18-e202501957-s001.pdf]

Tuning Crystal Growth of Colloidal $Cs_3Bi_2I_9$ Perovskite-like Nanocrystals via a Solvent-Assisted Reprecipitation Approach

Valentina Bellotti,[a] Francesca Pallini,[a] Sara Mattiello,[a] Charl Xavier Bezuidenhou,[a] Clara Saetta,[a] Giovanni Di Liberto,[a] Vanira Trifiletti,[a] Abdolhamid Khodadadi,[b] Gloria Zanotti,[c] Roberto Flammini,[b] Giorgio Contini,[b] and Luca Beverina*[a]

[a] Università degli Studi di Milano-Bicocca, Via Roberto Cozzi 55, 20126 Milano, Italy

[b] Istituto di Struttura della Materia-CNR (ISM-CNR), Via del Fosso del Cavaliere, 00133 Roma, Italy

[c] Istituto di Struttura della Materia-CNR (ISM-CNR), Strada provinciale 35d/9, 00010 Montelibretti, Italy

Materials and Methods

Materials

CsI (99.9%, Thermo Fisher), BiI3 (99.99%, Thermo Scientific) stored in glove box under argon, N,N-dimethylformamide anhydrous (DMF; 99.8%, Thermo Fisher), Dimethylsulfoxide anhydrous (DMSO; 99.7%, Acros), 1-Methyl-2-pyrrolidinone anhydrous (NMP; 99.5%, Acros), Triethyl phosphate (TEP; 98.0%, Alfa Aesar), Cyrene™ (Sigma-Aldrich), Toluene (VWR), 2-Butanone (99%, Sigma-Aldrich), Acetone (Technical grade, Carlo Erba), Benzonitrile (99%, Sigma-Aldrich), Butanol (BuOH; 99.4%, Fluorochem), Cyclohexane (99.8%, Carlo Erba), Dichloroethane (DCE; 99%, Sigma-Aldrich), Diethyl ether (Et_2O; Honeywell), Dimethyl carbonate (DMC; 99%, Sigma-Aldrich), N,N-dimethylacetamide (DMAc; 99%, Sigma-Aldrich), 1,4-Dioxane (99.5%, Thermo Fisher), Ethyl acetate (EtOAc; 99.9%, Honeywell), Methanol (MeOH; 99.9%, Carlo Erba), Methyl-t-Butyl Ether (MTBE; 98%, Sigma-Aldrich), N-methylimidazole (NMI; 99%, Sigma-Aldrich), Oleic Acid (OA; 90%, Alfa Esar), Propionic Acid (PA; 99.5%, Sigma-Aldrich), Tetramethyl guanidine (TMG; 99%, TCI), Tetrahydrofuran (THF; Carlo Erba), Triethylamine (TEA; 99%, Acros), Tributylamine (TBAm; 99.5%, Fluka), Deuterated chloroform (CDCl3; 99.8%, Eurisotop). All materials were used as received without further purification. A turbo-emulsifier homogenizer was bought from IKA and is composed of a motor group T25 digital Ultra Turrax + dispersing tool with codes S25N-25G for volumes up to 2 L.

Experimental section

Synthesis of $Cs_3Bi_2I_9$ nanocrystals. In a standard procedure 106.1 mg (0.18 mmol) of BiI_3 are dissolved in 6 mL of the selected solvent under argon atmosphere in a glove box and stirred upon complete dissolution (except for Cyrene™ in which the powder is not completely solubilized). Then, 70.15 mg (0.27 mmol) of CsI are added to the prepared solution and stirred for 1 hour. Only for the experiment in reducing environment, 2.6 mg (0.02 mmol) of phenyl hydrazine is added. The precursor solution was taken out of the glovebox and swiftly injected into 150 mL of toluene under vigorous stirring (900 rpm). After 30 seconds the resulting dispersion was centrifuged at 4500 rpm for 2:30 minutes. The precipitate was washed three times with toluene to remove any unreacted species and solvent traces. The powder was dried under vacuum for 2 hours and kept under static vacuum overnight in dryer.

Scale up of $Cs_3Bi_2I_9$ nanocrystals from DMF. The procedure described below has been scaled up, targeting 1g of final compound. In particular, 711.2 mg (1.2 mmol) of BiI_3 are dissolved in 40 mL of DMF under argon atmosphere in a glove box and stirred upon complete dissolution. Then, 470 mg (1.81 mmol) of CsI are added to the prepared solution and stirred for 1 hour. The precursor solution was taken out of the glovebox and swiftly injected into 1 L of toluene using a turbo-emulsifier (speed rate 13) to ensure optimal homogenization as previously reported for $CsPbBr_3$ nanocrystals.[8,24] After 30 seconds the resulting dispersion was centrifuged at 4500 rpm for 2:30 minutes. The precipitate was washed three times with toluene to remove any unreacted species and solvent traces. The powder was dried under vacuum for 2 hours and kept under static vacuum overnight in dryer. The final yield is 86%

Synthesis of ligand-stabilized $Cs_3Bi_2I_9$ nanocrystals. In a standard procedure 35.4 mg (0.06 mmol) of BiI_3 are dissolved in 2 mL of DMF under argon atmosphere in a glove box and stirred upon complete dissolution. Then, 23.38 mg (0.09 mmol) of CsI are added to the prepared solution and stirred for 1 hour. The precursor solution was taken out of the glovebox and swiftly injected into 50 mL of a toluene oleic acid (OA) mixture (3 mL of OA) under vigorous stirring (900 rpm). After 30 seconds the resulting dispersion was centrifuged at 4500 rpm for 2:30 minutes. Both the supernatant and precipitate were collected. The powder was dried under vacuum for 2 hours and kept under static vacuum overnight in dryer.

Computational details

We performed density functional theory calculations as implemented in the VASP[49] simulation package. The exchange-correlation functional was accounted by the Perdew-Burke-Ernzerhof (PBE) parametrization.[50] This choice can be considered acceptable due to the cost of

simulations, especially for those accounting for cluster formation, see below. All calculations include spin polarization. Dispersion interactions were accounted by means of Grimme's D3 scheme.[51] Valence electrons were expanded on a set of plane waves with a working kinetic cutoff equal to 400 eV, whereas core electrons were treated by Plane Augmented Wave (PAW) pseudopotentials.[52,53] The sampling of the reciprocal space was adopted to provide converged results. We decided to neglect Spin Orbit Coupling (SOC) due to specific purpose the study, which focuses on the structure, surface energy, and reactivity of surfaces. However, it must be mentioned that SOC affects the electronic structure of the system.[54-56] The inclusion of SOC would be computationally not affordable. This can be considered an acceptable choice if the focus is not directly on the nature of the electronic excitation of the material. We started from the experimental bulk crystal structure and optimized it. Then, we designed (0001) surface models with different terminations. Then, we considered the formation of Bi clusters. To do so, we invoked larger simulation cells, as detailed below in the text. In the case of Bi_4, the working simulation box contained 384 atoms.

Characterization techniques

UV-visible spectroscopy in solution. Light absorption at specific wavelength in solution have been measured through a double beam Jasco V-570 UV-visible absorption spectrophotometer with scan rates of 200 nm/min. The CBI samples have been dispersed in the selected solvent with initial concentration of 1 mg/mL and then diluted to overcome saturation. For the measurements of BiI_3 and BiI_3/CsI clusters the concentration for the measurement was the same used for the synthesis. The wavelength range used in all measures was 350-700 nm.

UV-visible spectroscopy with integrated sphere. Crystals' ability to absorb light was evaluated by spreading them on a glass substrate and measuring their spectra through a Jasco V-770 spectrometer equipped with an integrating sphere (Jasco ISN-470).

Dynamic Light Scattering (DLS). Hydrodynamic volume and size distribution of nanocrystals were determined by dynamic light scattering. The measures were recorded at 25 °C on a Malvern Zetasizer Nano S equipped with a continuous wave 1mW He-Ne monochromatic laser operating at 632.8 nm and an avalanche photodiode detector, Q.E. > 50% at 633 nm, placed at 173° with respect to the incident beam. Reported data are the average of three different measurements of the size distribution as the function of the intensity.

X-ray diffraction PXRD experiments. PXRD were performed on a Rigaku SmartLab powder diffractometer using Cu-Kα radiation, 40 kV, 30 mA over a range for 2θ of 5.0 - 60.0° with a step size of 0.01° and a scan speed of 2.0°·min^{-1} at room temperature in air.

Rietveld Refinement. Rietveld structural refinements of the X-ray data were performed using the TOPAS-Academic64 V6 software package.[57,58] The initial model was obtained from the literature and the atomic coordinates refined taking into consideration the special positions of the atoms. The structure was modelled and refined in the *P*63/*mmc* space group. The background was fitted and refined using a Chebyshev polynomial with 10 coefficients in the range from 5° to 60° 2-theta with baseline shift refinement. The "Simple_Axial_Model" accounted for the asymmetry in the peaks, especially at low 2-theta values. The peaks were fitted using a PearsonVII " PVII " function. Additional corrections include March-Dollase preferred orientation corrections on the ((0 0 6), March parameter (r) – 0.2959) and ((1 0 1), March parameter (r) – 0.43499) reflections for the sample form-DMSO which shows a high degree of preferred orientation along the *c*-axis. The following equation estimates the degree of preferred orientation within the March–Dollase approach.[59,60]

$$\eta = 100\% \left[\frac{(1-r)^3}{1-r^3}\right]^{1/2} \qquad (1)$$

Where η is the percentage degree of preferred orientation and r is the March parameter. This yields a degree of preferred orientation of 60% and 44% for (0 0 6) and (1 0 1) respectively.

The particle-size (Integral breadth based LVol) for the compounds were evaluated by Rietveld refinement (better evaluation of the diffraction intensities compared to Pawley refinement) using the size-strain double-Voigt approach, as implemented in the TOPAS academic software.[35]

Nuclear magnetic resonance (NMR). The chemical composition and contaminants of solvents were investigated through ^{1}H NMR, recorded using a Bruker Ascendent 400 spectrometer operating at 400 MHz, equipped with a 9.4T magnet. The NMR tube was prepared with few drops of the same solvent baches used for the synthesis, dissolved in 0.7 mL of deuterated chloroform $CDCl_3$.

Transmission electron microscopy (TEM). TEM images on materials were collected by using a JEOL JEM-2100Plus TEM (JE OL, Akishima, Tokio, Japan) operating at an acceleration voltage of 200 kV, equipped with an 8-megapixel Gatan (Gatan, Pleasanton, CA, USA) Rio complementary metal-oxide-semiconductor camera. The CBI samples were prepared by drop casting a toluene dispersion (~ 0.1 mg/mL) onto carbon-coated Cu TEM mesh grids. High

resolution imaging was performed in parallel illumination mode using a CMOS Gatan RIO camera.

Scanning electron microscopy (SEM). The analysis was performed using a Tescan VEGA TS5136XM scanning electron microscope equipped with energy-dispersive X-ray spectroscopy (SEM-EDX). The CBI samples were prepared by spin coating a toluene dispersion (~ 10 mg/mL) onto glass. The latter was attached to the SEM stab using carbon tape. Gold was deposited onto the samples through a metal evaporator prior analysis.

X-ray photoelectron spectroscopy (XPS). XPS studies were performed using an ultra-high vacuum (UHV) instrument equipped with aluminum Kα X-ray source (hυ=1486.6 eV) and a five-channeltron (VG 150 mm mean radius) hemispherical electron analyzer. The spectra were analyzed by fitting to the data using Voigt functions and a Shirley background. The XPS data elaboration has been partially performed using CONTUR.[61] During the XPS measurements, a charging effect was observed. To correct this, the C1s binding energy (BE) at 284.6 eV was used as a reference[62] and all core levels were shifted accordingly. All data were then normalized to the intensity at lower binding energy. The data have been fitted considering a spin-orbit splitting of 5.3 eV for Bi 4f.[63] The expected area ratio for Bi 4f 7/2 to 4f 5/2 was set to 4/3.[64] During the fitting, an additional component was needed to account for the spectral features, corresponding to Cs 4p3/2 and overlapping with the Bi 4f4/2 peak at 159.4 eV.[65,66] As for the C 1s fitting procedure, the most intense component was set at BE = 284.6 eV as a reference. Additional unknown components were found during the fitting. For O 1s, the same width and constraints were applied as for C 1s case.

Thermal gravimetric analysis (TGA). TGA analysis was carried out with a Mettler Toledo TGA/DSC STARe System, at a constant gas flow (50 cm^3/min). The thermal profile was the following: from 30 °C to 600 °C with heating rate of 10 °C/min under nitrogen flow. Alumina crucible was used as filled with around 10 mg of solid material.

Additional Data

S1: Solubility parameters

The solvents were initially classified according to Kamlet-Taft polarizability (π*) and hydrogen bond acceptor (β) parameters, Gutmann donor number (DN) and dielectric constant (ε) to evaluate both their capability to solubilize CsI and BiI_3, and to produce a stable dispersion of the obtained $Cs_3Bi_2I_9$ nanocrystals. We chose these polarity scales for the following reasons:

- Gutmann donor number measures the capability of a solvent to behave as a Lewis base (therefore being able to form complexes with Lewis acids, such as Bi^{3+} and Cs^+)
- The dielectric constant measures the capability of a material to store electrical energy when exposed to an electric field. Therefore, it can be used as a guideline to predict the capability of a solvent to dissociate ions of opposite charge (which is needed to solubilize ionic substances such as CsI and BiI_3)
- Kamlet-Taft parameters categorize solvents on the basis of their capability to behave as Lewis acids (α) or bases (β), and on their polarizability (π*).

DN and β are therefore different scales to measure solvents basicity according to Lewis, while ε and π* can both be used as guidelines to individuate good solvents for salts. We did not take into consideration Hildebrand and Hansen solubility parameters, as they are generally not suitable to predict solubility of ionic species.

Table S1: Solvents polarizability (π*), hydrogen bond acceptor ability (β), donor number (DN) and dielectric constant (ε) parameters from literature. If not otherwise stated, data source is [32] for π*, β and DN, and [67] for ε

Solvent	Abbreviation	β	π*	ε	DN
1-methyl-2-pyrrolidone	NMP	77	92	32.2	27.3
2-butanone	MEK	48	67	18.51	17.4
Acetone	Acetone	43	71	21.01	17
Benzonitrile	Benzonitrile	37	90	25.9	11.9
Butanol	BuOH	84	47	17.51	29
Cyclohexane	Cyclohexane	0	0	2.02	0
Cyrene	Cyrene	61	93	32.2	27
Dichloroethane	DCEt	10	48	10.36	0
Diethyl ether	Et_2O	47	27	4.33	19.2
Dimethyl carbonate	DMC	43	45	3.17	17.2

Dimethyl sulfoxide	DMSO	76	100	46.68	29.8
DMAc	DMAc	76	88	37.78	27.8
Dioxane	Dioxane	37	55	2.38	14.3
Ethyl acetate	AcOEt	45	55	6.02	17.1
Methanol	MeOH	66	60	32.7	30
Methyl-t-Butyl Ether	MTBE	49	27	4.5	18.1
N-methylimidazole	NMI	82	97	30	27.6
N,N-dimethylformamide	DMF	69	88	36.71	26.6
Propionic Acid	PA	45	58	3.1	20
Tetramethyl guanidine	TMG	86	76	23.1	31
THF	THF	55	58	7.58	20
Toluene	Toluene	11	54	2.38	0.1
Triethylamine	TEA	71	14	2.42	61
Triethyl phosphate	TEP	77	72	13.01	26
Tributylamine	TBAm	62	16	2.29	50
Water	Water	47	109	80.1	18

After a preliminary screening, we found that both β and DN correlate with the solubility of precursors. This result is not unexpected, as both CsI and BiI_3 behave as a Lewis acids, and therefore can be solubilized by solvents that are Lewis bases. We therefore decided to further investigate both precursors solubility and $Cs_3Bi_2I_9$ nanocrystals dispersibility using Kamlet-Taft parameters β and π*. DN scale in fact is known to correlate with β,[68] but DN values often suffer lack of precision due to the difficulty in measuring the enthalpy of formation of the $SbCl_5$-solvent complex.[69] Plotting β and π* within the same graph, we finally found that both parameters are important to correctly define solvents capable to dissolve the precursors, and to produce stable dispersions of the final CBI nanocrystals (i.e. solvents that do not dissolve back the CBI).

S1.2 Stability of $Cs_3Bi_2I_9$ nanocrystals

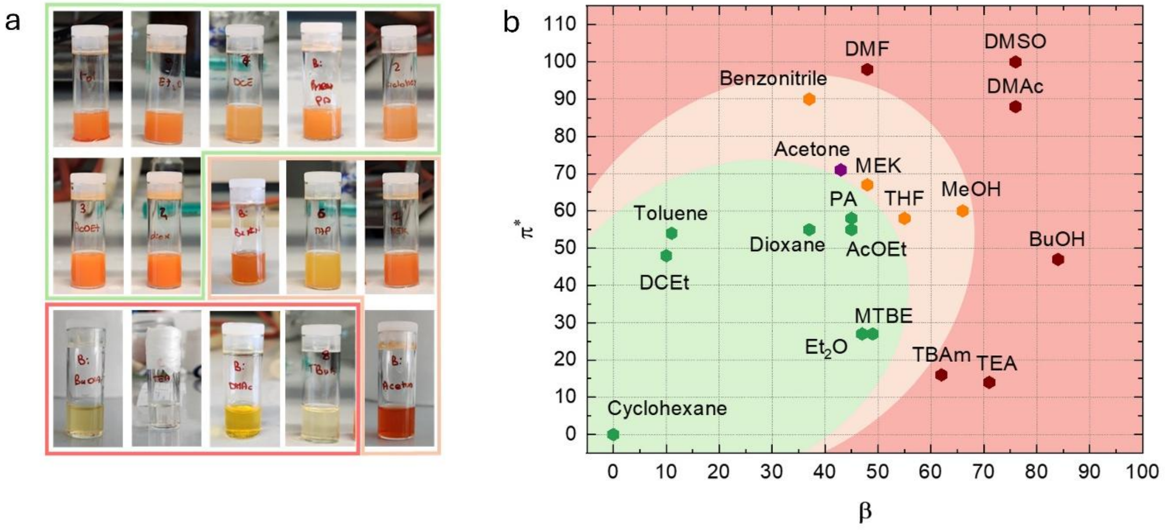

Figure S1: On the left, picture of CBI dispersions marked with green, orange and red squares depending on the solvent position in the right panel. Acetone have a peculiar behavior, as it efficiently disperses the nanocrystals, but they remain stable for around 12 hours before forming a yellow precipitate. On the right, solubility plot representing Kamlet-Taft polarizability (π*) vs hydrogen bond acceptor ability (β).The red region includes solvents that destroyed CBI nanocrystal at 1 mg/mL concentration, providing UV-vis spectrum of the precursors. Inside the orange region CBI nanocrystals are stable for few hours or do not survive to dilution. The green region includes the solvents which results in stable CBI dispersion. All test have been performed on CBI synthetized from DMF.

S1.3 Solubility of precursors

The graph representing precursors solubility in different solvents is reported in the main text (fig. 1a). For the five solvents found to solubilize both CsI and BiI_3, UV-Vis absorption spectra were collected.

1 mg/mL of nanocrystals have been dispersed in different solvents and further diluted for the absorption measurements.

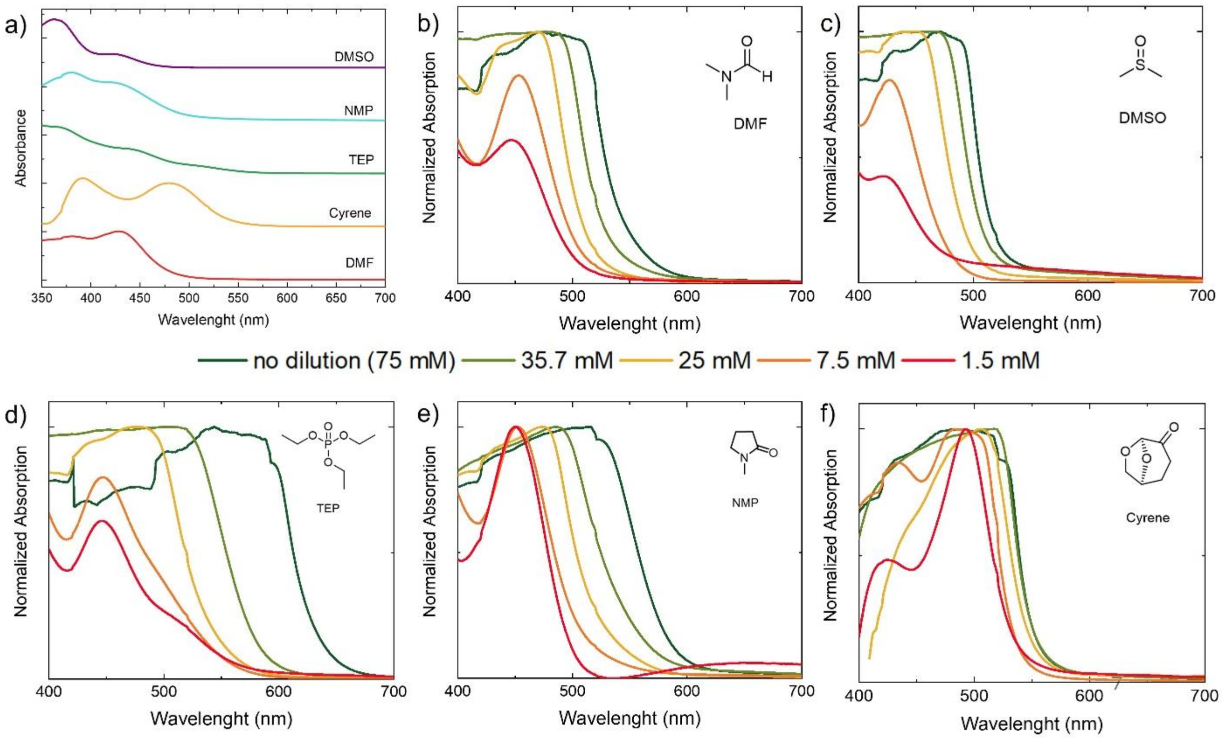

Figure S2: a) UV-visible absorption spectra of BiI_3 in DMF, DMSO, TEP, NMP and cyrene. Study of concentration dependency on BiI_3/CsI in b) DMF, c) DMSO, d) TEP, e) NMP and f) cyrene.

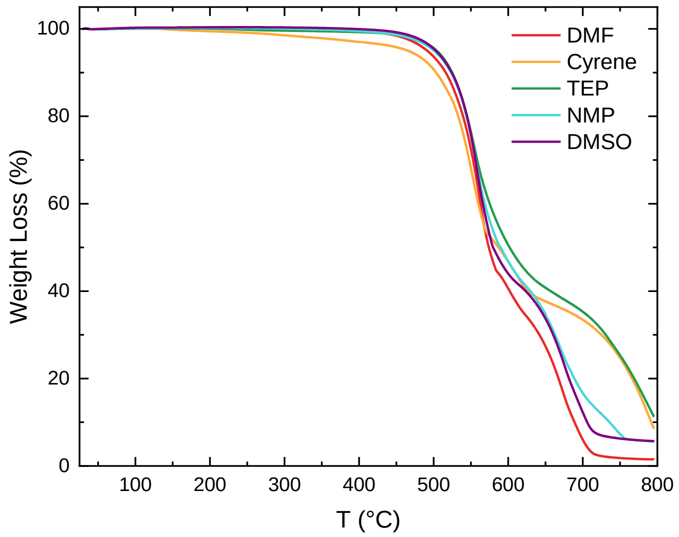

Figure S3: TGA analysis of CBI synthetized in different solvents.

S2: Dimension and morphology of the CBI crystals

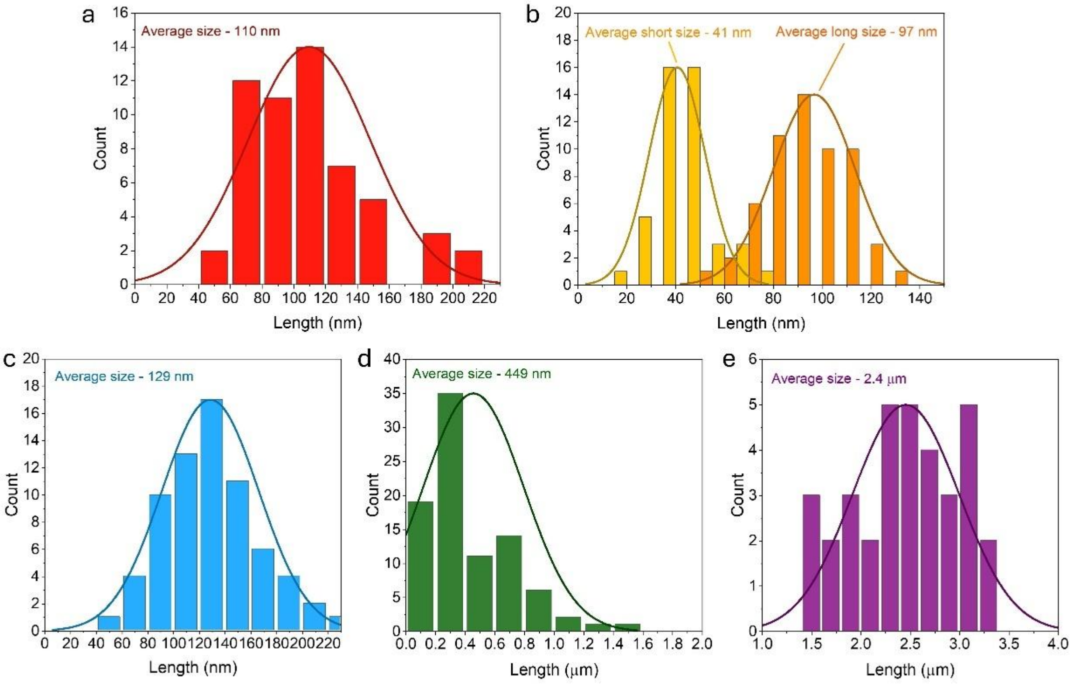

Figure S4: Dimensional Analysis of TEM images for CBI synthetized from a) DMF, b) Cyrene, c) NMP, d) TEM, and e) DMSO

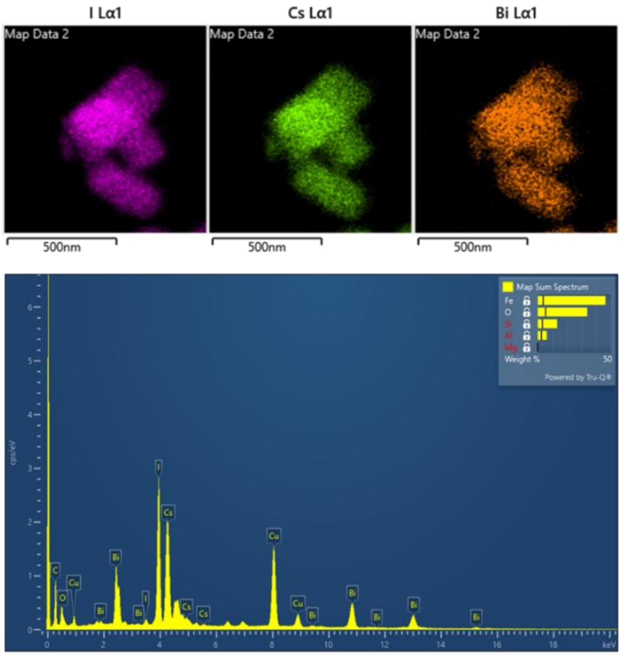

Figure S5: EDX analysis of nanocrystals synthetized from DMF.

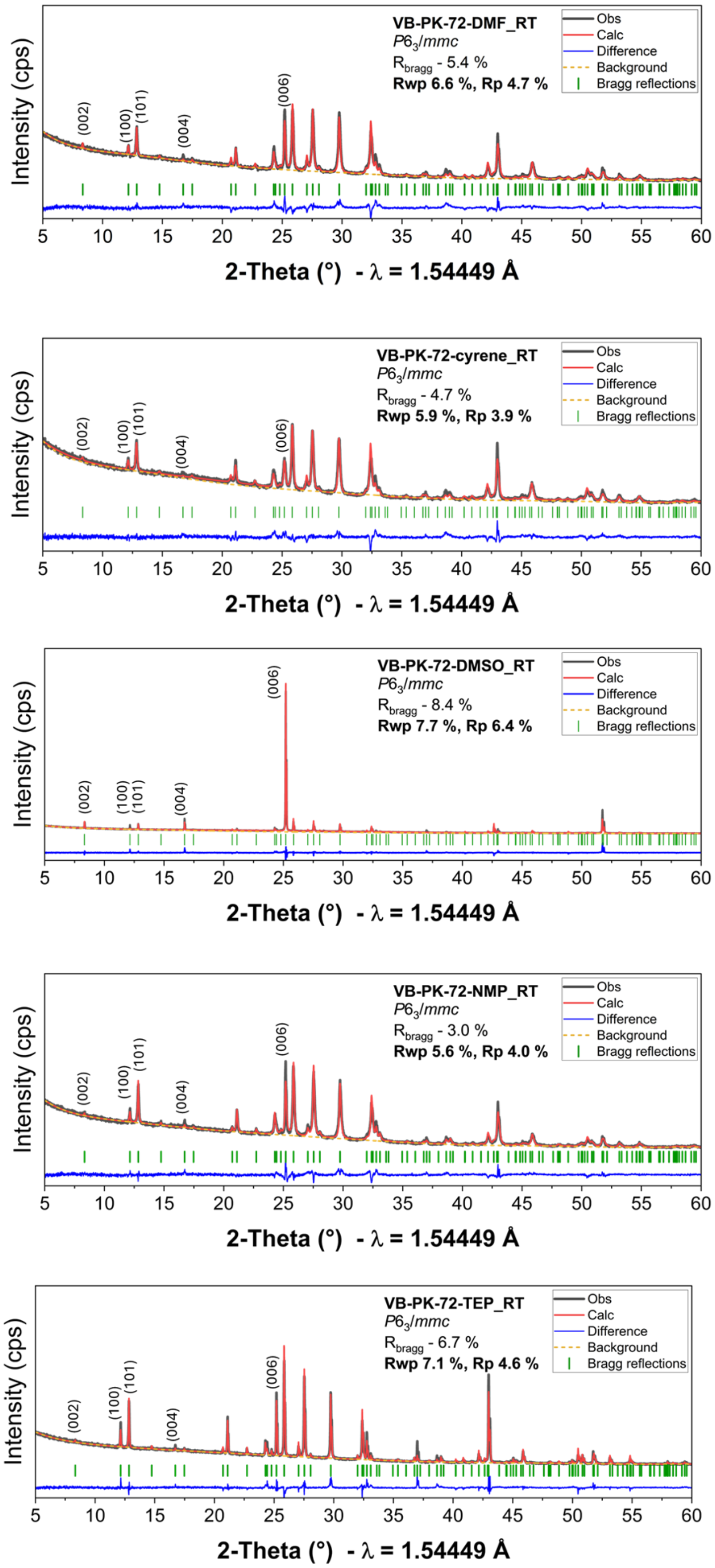

Figure S6: The Rietveld refinement plots for the samples grown from DMF, Cyrene, DMSO, NMP and TEP. The XRD data were collected at RT using the Rigaku SmartLab instrument.

Table S2: The refinement details and cell parameters for the Rietveld refinement for data collected at RT using the Rigaku SmartLab instrument.

	From DMF	From Cyrene	From NMP	From TEP	From DMSO
**Chemical Formula**	I9 Cs3 Bi2	I9 Cs3 Bi2	I9 Cs3 Bi2	I9 Cs3 Bi2	I9 Cs3 Bi2
**Formula Weight**	1958.81	1958.81	1958.81	1958.81	1958.81
**Z**	2	2	2	2	2
**Temperature**	298 K	298 K	298 K	298 K	298 K
**Space Group**	$P6_3/mmc$	$P6_3/mmc$	$P6_3/mmc$	$P6_3/mmc$	$P6_3/mmc$
**a/Å**	8.4079(6)	8.4101(7)	8.4097(4)	8.4104(2)	8.4120(2)
**b/Å**	8.4079(6)	8.4101(7)	8.4097(4)	8.4104(2)	8.4120(2)
**c/Å**	21.185(2)	21.194(2)	21.191(1)	21.1856(4)	21.1865(4)
**α/°**	90	90	90	90	90
**β/°**	90	90	90	90	90
**γ/°**	120	120	120	120	120
**V ($Å^3$)**	1297.0(2)	1298.2(3)	1297.9(2)	1297.82(6)	1298.35(6)
**ρ (g/cm^3)**	5.0155(8)	5.011(1)	5.0121(6)	5.0125(2)	5.0105(2)
**Average grain size (nm)**	71(4)	49(2)	130(13)	N.A. large crystallites	N.A. large crystallites
**Micro-strain (%)**	0.015(7)	0.075(8)	0.045(7)		
**Rbragg (%)**	5.4	4.7	3.0	6.7	8.4
**Rp (%)**	4.7	3.9	4.0	4.6	6.4
**Rwp (%)**	6.6	5.9	5.6	7.1	7.7
**Refinement Method**	Rietveld	Rietveld	Rietveld	Rietveld	Rietveld
**Measurement Device Type**	Rigaku SmartLab	Rigaku SmartLab	Rigaku SmartLab	Rigaku SmartLab	Rigaku SmartLab
**Radiation Wavelength (Å)**	1.544493	1.544493	1.544493	1.544493	1.544493
**Measurement Method**	Continuous Scan	Continuous Scan	Continuous Scan	Continuous Scan	Continuous Scan

S3: Colloidal stability

The colloidal stability of CBI synthetized via SARP and LARP methods have been tested. 0.5 mg of nanocrystals have been suspended in 1 mL of toluene, sonicated for 20 minutes and added into a four optical quartz cuvette. Both DLS and absorption measurements have been performed at t_0, after 1 hour, 1 day and 3 days.

For the SARP method, samples derived from DMF and DMSO have been selected as relevant examples of nanocrystals with very different Bi^0/Bi^{3+} ratio, according to XPS data (**Figure 4** in the main text). As expected, the CBI synthetized from DMSO have very poor colloidal stability. In fact, sedimentation occurs after few minutes. On the other hand, CBI synthetized form DMF remains suspended for 1 day, whereas complete sedimentation occurs after 3 days, confirming the higher colloidal stability.

SARP has been compared with a common LARP method, using a large excess of oleic acid (OA) as ligand. Often in LARP procedure, after the injection of the precursor's solution in the antisolvent and centrifugation, the precipitate is discharged and only the supernatant is stocked. Due to the very high amount of material recovered after centrifugation, we decided to test the colloidal stability of both the powder and the supernatant. It is clear from **figure S7b** that, at equal weight concentration, the amount of CBI is different, suggesting a larger amount of OA in the supernatant. Despite the ligand stabilization both the supernatant and the powder produced by LARP sedimented after 16 hours. From the DLS it is possible to appreciate that at time zero different size populations are present but as sedimentation occurs only the smaller particles remain suspended.

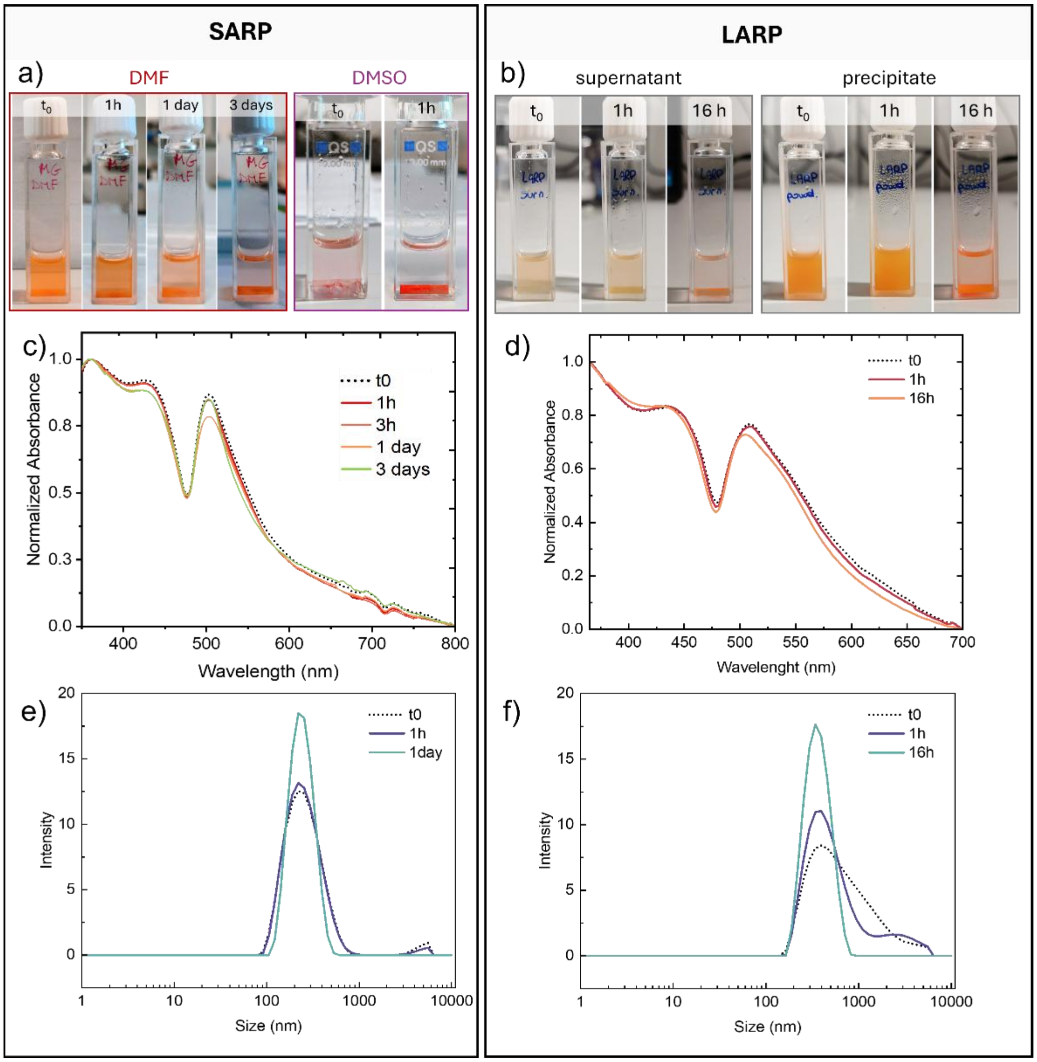

Figure S7: Stability measurements, including pictures of (a) SARP samples from DMF and DMSO and (b) LARP sample both the supernatant and the recovered powder. UV-visible absorption and DLS of CBI crystals synthetized *via* (c,e) SARP and (d,f) LARP. In the right panel only the measurements for precipitate are reported due to the very low absorbance of the supernatant dispersion.

S4: X-ray photoelectron spectroscopy (XPS)

The survey scan (Fig. S5) revealed the primary elements present, including bismuth (Bi 4f), iodine (I 3d), and cesium (Cs 3d), along with carbon (C 1s), nitrogen (N 1s), and oxygen (O 1s).

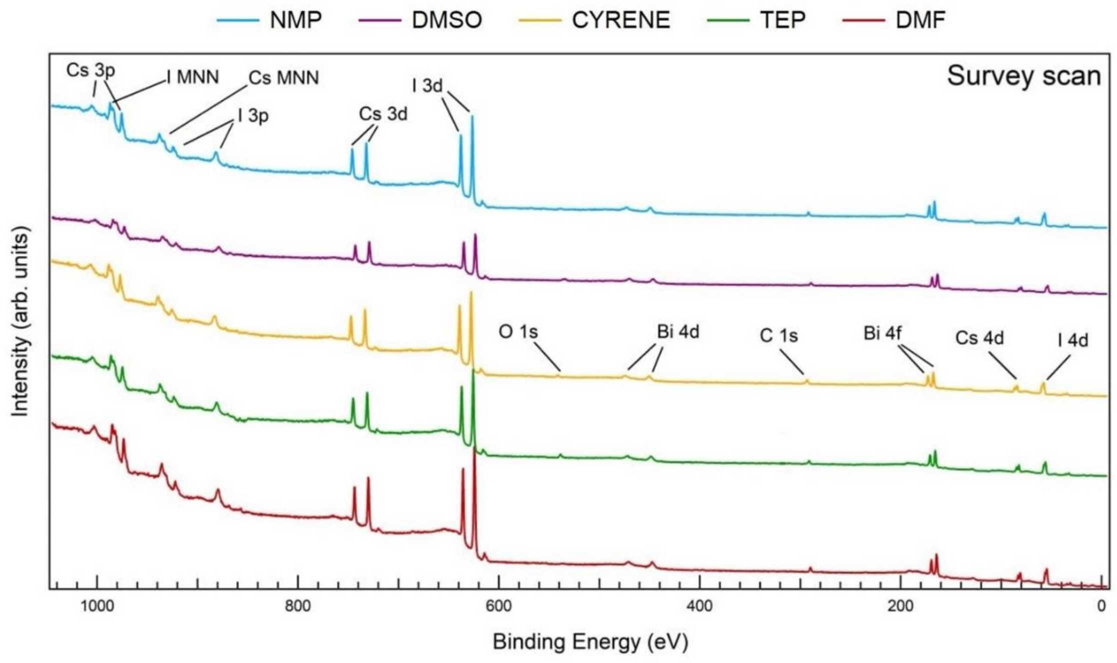

Figure S8: XPS Survey scan spectra of $Cs_3Bi_2I_9$ processed from different solvents including NMP, DMSO, Cyrene, TEP, and DMF.

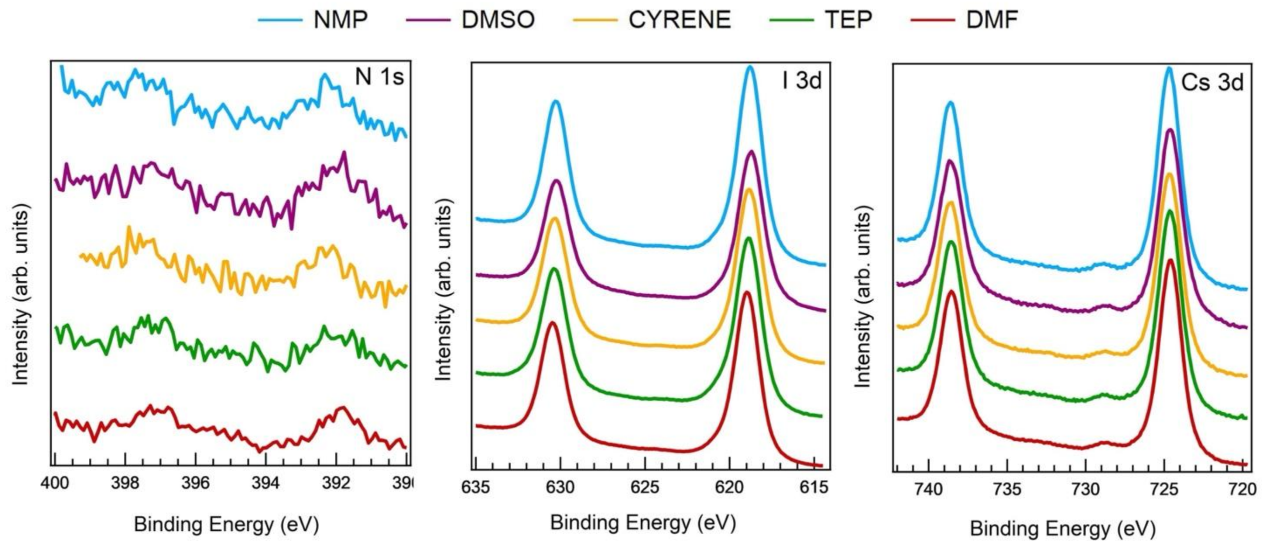

Figure S9: Core level XPS spectra of N 1s, I 3d, and Cs 3d regions for $Cs_3Bi_2I_9$ processed from different solvents including NMP, DMSO, Cyrene, TEP, and DMF.

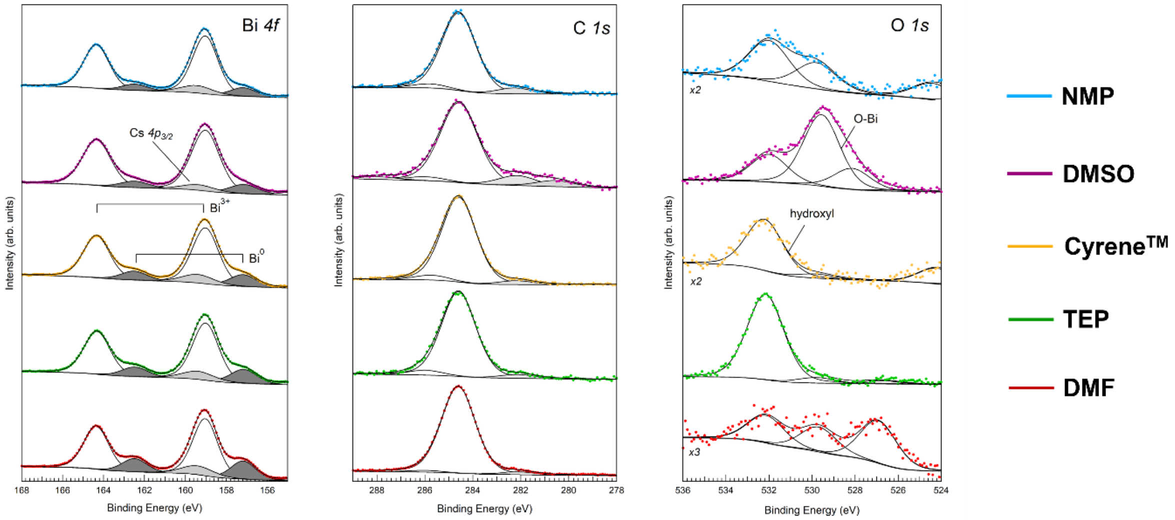

Figure S10: Core-level XPS spectra of the Bi 4f, C 1s, and O 1s regions for $Cs_3Bi_2I_9$ films processed using NMP, DMSO, Cyrene, TEP, and DMF. Spectra are shown along with their corresponding peak fittings to highlight compositional and chemical differences. The data are arranged from top to bottom in order of increasing Bi^0 contribution, reflecting the influence of the processing medium on the material's chemical composition.

Table S3: Binding energy values, deconvoluted peak areas, and subpeak ratios of core-level XPS spectra (N 1s, I 3d, Cs 3d, and Bi 4f) for $Cs_3Bi_2I_9$ processed from five different solvents: NMP, DMSO, Cyrene, TEP, and DMF. The calculated areas under each core-level peak and subpeak are reported, along with their relative ratios. Elemental area ratios with respect to Bi 4f, and the Bi 4f subpeak area ratios (Bi^{3+}/Bi^0 or other species).

**Binding energy (eV)**					
	DMF	NMP	TEP	DMSO	CYRENE
$Bi^{3+}_{7/2}$	159.0	159.0	159.0	159.0	159.0
$Bi^{3+}_{5/2}$	164.3	164.3	164.3	164.3	164.3
$Bi^{0}_{7/2}$	157.2	157.2	157.2	157.2	157.2
$Bi^{0}_{5/2}$	162.5	162.5	162.5	162.5	162.5
Cs 4p	159.4	159.4	159.4	159.4	159.4

Table S4: Calculated area under peaks and subpeaks, and their relative ratio.

**Calculated area under peaks**					
C1s	1.08	0.71	0.71	0.60	0.97
O1s	0.07	0.14	0.37	0.45	0.13
Bi4f	4.57	4.79	4.24	6.03	4.10
Cs 3d	2.73	2.86	2.72	2.69	2.79
I 3d	10.23	11.30	10.64	9.28	10.19

**Subpeak Area**					
C-C	1.02	0.64	0.65	0.46	0.88
hydroxyl	0.02	0.07	0.31	0.10	0.09
Bi-O	0.02	0.04	0.02	0.27	0.01
**Area ratio elements regard to Bi4f**					
C 1s / Bi 4f	0.24	0.15	0.17	0.10	0.24
O 1s / Bi 4f	0.01	0.03	0.09	0.07	0.03
Cs 3d / Bi 4f	0.60	0.59	0.64	0.44	0.68
I 3d / Bi 4f	2.24	2.36	2.51	1.54	2.48
Cs 3d/ I 3d	0.27	0.25	0.25	0.29	0.27
**Area ratio Bi subpeaks**					
$Bi^{3+}_{7/2}$/ $Bi^{0}_{7/2}$	3.14	6.72	4.12	6.53	4.74
$Bi^{3+}_{7/2}$/Cs4p	5.04	6.20	5.78	9.38	5.43

S5: NMR Analysis of commercial solvents

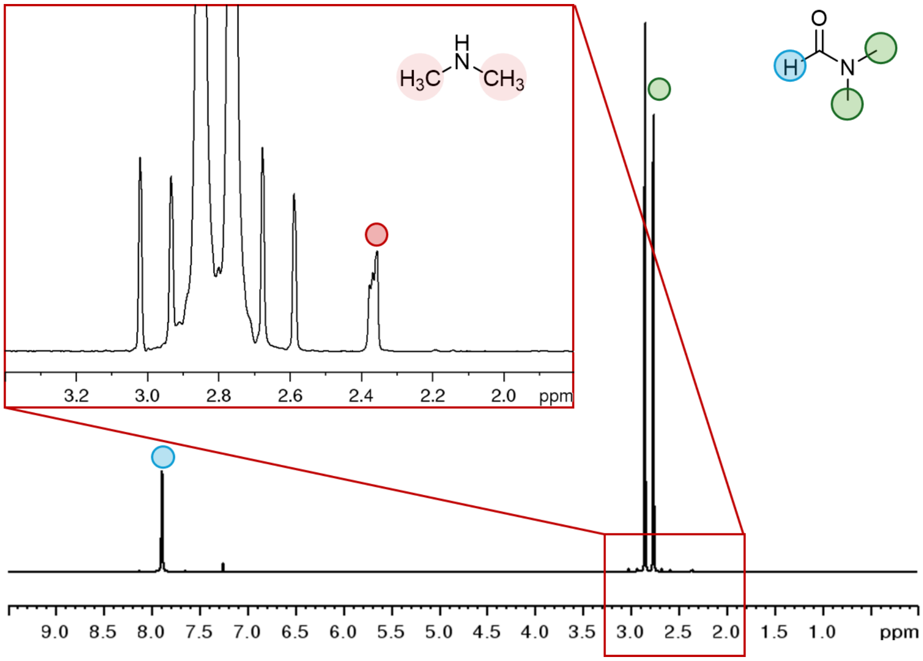

Figure S11: ^{1}H NMR of anhydrous DMF in $CDCl_3$. Quantification of DMA byproduct by integration is 0.4 mol%.

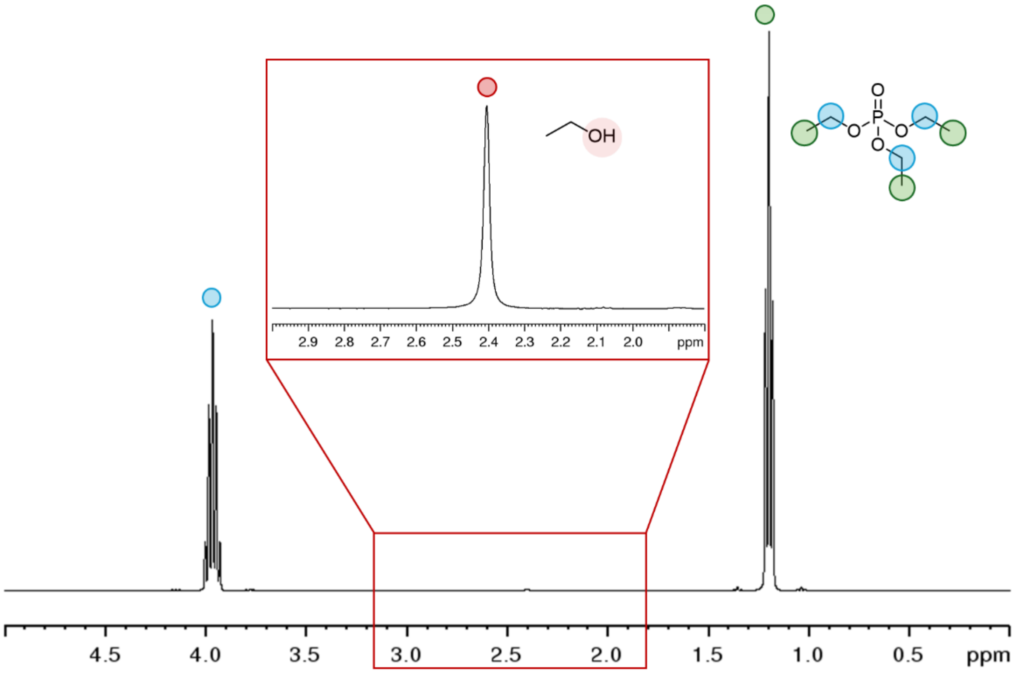

Figure S12: ^{1}H NMR of anhydrous TEP in $CDCl_3$. EtOH is present as a byproduct.

<u>*S6: Density Functional Theory*</u>

S6.1 Evaluation of surface models

The optimized lattice parameters for the bulk cell are: $a = b = 8.470$ Å, $c = 21.631$ Å with $\gamma = 120°$, in accordance with experimental. To design the surface models, we considered the low index surface (0001), considering the experimental evidence of hexagonal crystal growth. The (0001) surface can be terminated as depicted in Figures S10a-c. The models were constructed to have non-polar surfaces. The names assigned to the different terminations refer to the atoms available on the surface. However, different species can be slightly less exposed but still can interact at the interface (eg. I_3-term in *panel b* also exposes Cs atoms). Table S5 reports the calculated lattice vectors, thickness of the model and surface energy.

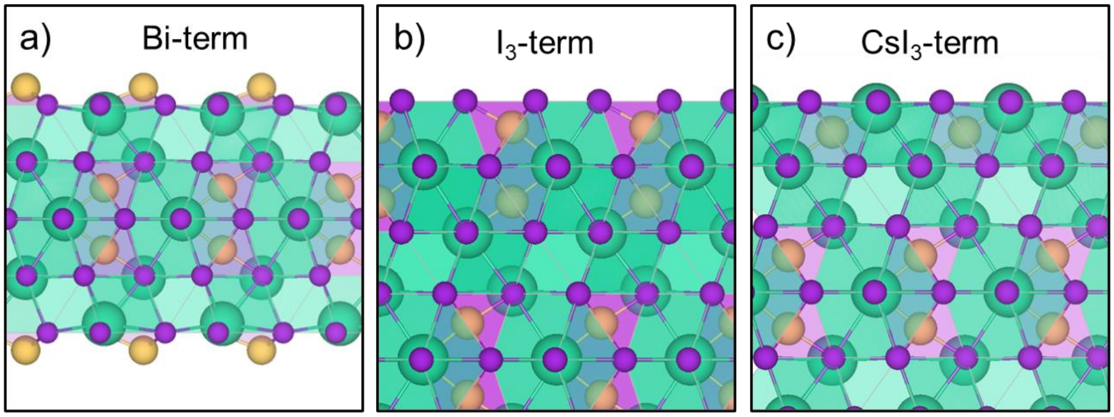

Figure S13: (0001) possible termination with different exposed atoms for $Cs_3Bi_2I_9$ surface.

Table S5: calculated lattice vectors, thickness of the model and surface energy of $Cs_3Bi_2I_9$

	Termination	***a /Å***	***b / Å***	***γ / °***	***d / nm***	***E_s / J/m²***
001	Bi-term	8.470	8.470	120.0	1.6	0.48
	I_3-term	8.470	8.470	120.0	2.1	0.47
	CsI_3-term	8.470	8.470	120.0	2.8	0.65

S6.2 DMA interaction with the different surface models

The presence of Bi and Cs atoms on the surface can promote chemical interaction between the perovskite and the solvent. When DMF is considered as the solvent DMA byproduct is formed. To consider a reasonable coverage we worked with 2x2 expansions of the unit cells. We evaluate the interaction of DMA with the surface. Figures S11a-b shows the interaction of the DMA nitrogen lone pair and the two metals. If the DMA adsorption occurs on Bi, the bond lengths are around 2.60 Å. If the DMA binds Cs a pyramidal coordination of the metal is found with larger bond lengths, around 3.00 Å.

$$\Delta E_{ads,Bi-DMA} = -4.27\ eV$$

$$\Delta E_{ads,Cs-DMA} = -2.78\ eV$$

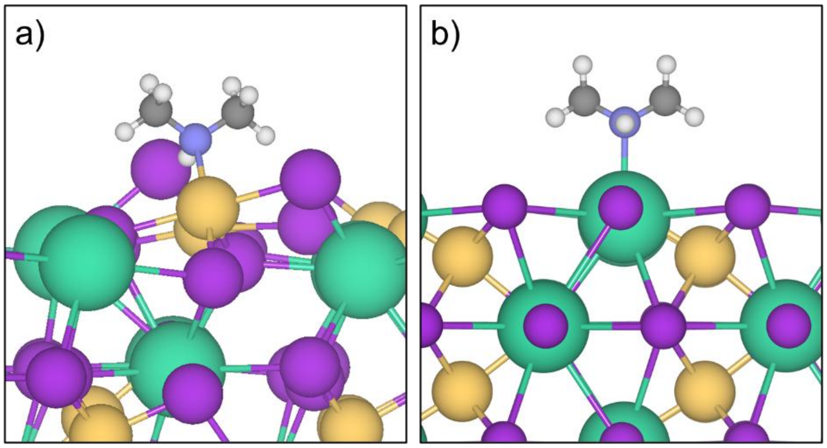

Figure S14: *(*a) DMA adsorbed on a Bi site; (b) DMA adsorbed on a Cs site of $Cs_3Bi_2I_9$ (0001).

S6.3 Bi_n clusters formation

The possible formation of Bi aggregates on the surface of the material was considered, with consequent oxidation of solvent molecules. The surface chemistry of clusters is strongly dependent on the size and morphology of the aggregate. For this reason, we simulated the formation of clusters of increasing size to check for the consistency of the results. The formation of Bi clusters can occur from the aggregation of Bi atoms on the surface. To do so, we generated a large supercell of the (0001) Bi-terminated surface, with $a = b = 33.880$ Å, to have enough surface Bi species. The number of surface Bi atom is 32, therefore we decided to simulate the formation of Bi_n with $n = 8$ at most. The reaction free energies for the cluster formation normalized by the number of (Bi) atoms converges from $n = 4$.

S7: Scale up

As described in the experimental section the synthesis of CBI nanocrystals from DMF precursor solvent has been scaled up targeting 1g of final material using a turbo-emulsifier for homogenization. The resulting material has been characterized both *via* XRD, and spectroscopy as reported in **figure S15**.

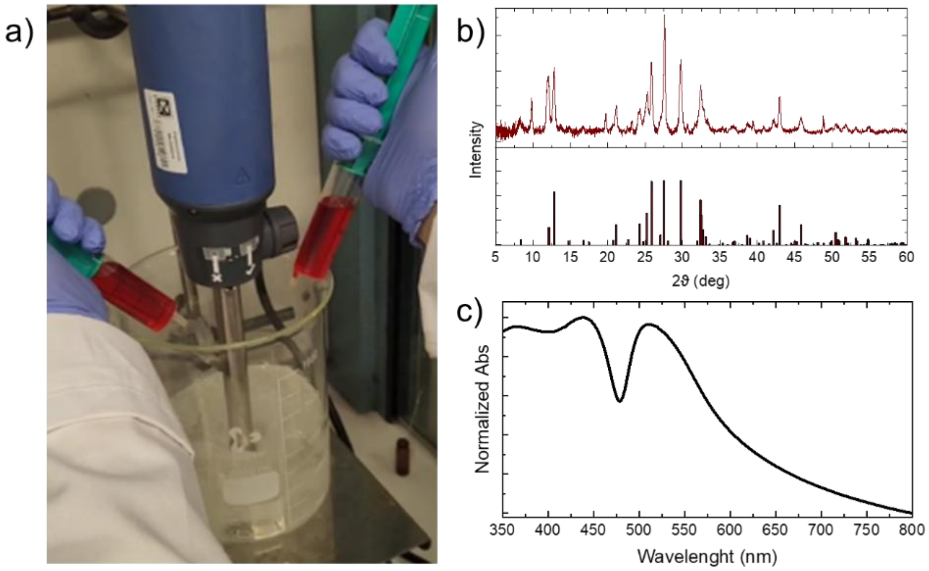

Figure S15: *(*a) photograph of the scaled-up synthesis of CBI nanocrystals using a turbo-emulsifier; (b) XRD and (c) UV-vis absorption of the resulted nanocrystals.
